# Utility of serological biomarker’ panels for diagnostic accuracy of interstitial lung diseases

**DOI:** 10.1007/s12026-020-09158-0

**Published:** 2020-10-22

**Authors:** Laura Bergantini, Miriana d’Alessandro, Lucia Vietri, Giuseppe Domenico Rana, Paolo Cameli, Silvia Acerra, Piersante Sestini, Elena Bargagli

**Affiliations:** 1 Department of Medical Sciences, Surgery and Neuroscience, Respiratory Disease and Lung Transplant Unit, Siena University, Siena, Italy; 2 Internal Medicine Unit, Department of Internal Medicine, Geriatrics and Rare Diseases Unit, Bari University, Bari, Italy

**Keywords:** Biomarkers, Diagnosis, ILD, SAA, KL-6

## Abstract

Interstitial lung diseases (ILD) are a heterogeneous group of illnesses of known and unknown aetiology. Differential diagnosis among the three disorders is often challenging. Specific biomarkers with good sensitivity and specificity are therefore needed to predict clinical outcome and guide clinical decisions. The aim of this study was to investigate inflammatory/fibrotic biomarkers, to determine whether single mediators or panels of mediators could be useful to stratify patients into three distinct domains: sarcoidosis, idiopathic pulmonary fibrosis (IPF) and chronic hypersensitivity pneumonitis (cHP). A total of 163 ILD patients monitored at Siena Referral Centre for Sarcoidosis and other Interstitial Lung Diseases were enrolled in the study. Clinical data, pulmonary function tests and biochemical analytes were retrospectively collected. SAA levels were detected by ELISA kit and Krebs von den Lungen 6 (KL-6) were measured by CLEIA method, for sarcoidosis, cHP and IPF patients. Multiple comparison analysis showed significant differences in C reactive protein (CRP), white blood cell count (WBC) and creatinine levels between the three groups. In the logistic regression model, KL-6, CRP and WBC showed areas under curves (AUC) 0.86, for sarcoidosis diagnosis. The logistic regression model KL-6 and SAA showed the best performance with an AUC 0.81 for discriminating IPF than cHP and sarcoidosis. For differential diagnosis of IPF and cHP, KL-6 and SAA were considered in the logistic regression model, showed an AUC 0.79. The combination of serum biomarkers proposed here offers insights into the pathobiology of ILDs. These panels of bioindicators will improve diagnostic accuracy and will be useful in the clinical management of ILDs.

## Introduction

Interstitial lung diseases (ILD) are a heterogeneous group of illnesses of known and unknown aetiology, characterized by progressive fibrotic involvement of the lungs [1, 2]. They include sarcoidosis, chronic hypersensitivity pneumonitis (cHP) and idiopathic pulmonary fibrosis (IPF) [3]. Sarcoidosis is a multisystemic granulomatous disease, associated with T lymphocyte and macrophage activation and migration into affected organs [4–6]. The course of sarcoidosis is unpredictable: remission occurs in most cases with good outcomes, whereas persistent granuloma inflammation may lead to fibrotic lung disease [7, 8]. Chronic hypersensitivity pneumonitis is a fibrotic interstitial lung disease known to be caused by inhalation of different substances and chemical compounds that determine an inflammatory and immunological response in sensitized individuals [9, 10]. Idiopathic pulmonary fibrosis (IPF) is a chronic progressive fibrotic interstitial lung disease of unknown aetiology, characterized by an imaging and histological pattern known as usual interstitial pneumonia (UIP) [11, 12].

Differential diagnosis between the three disorders is often challenging. Specific biomarkers with good sensitivity and specificity are therefore needed to predict clinical outcome and guide clinical decisions [13–17]. Krebs von den Lungen 6 (KL-6) and serum amyloid A (SAA) have been widely studied and proposed as diagnostic and prognostic biomarkers of ILDs. KL-6 is a mucin-like glycoprotein, overexpressed in alveolar epithelial cells of ILD patients. [17–20]. Elevated KL-6 level seemed provide information for identifying patients with ILDs who are at increased risk for subsequent mortality [21]. Serum amyloid A (SAA) is an acute-phase protein with multiple immunological functions involved in lipid metabolism, granuloma formation and carcinogenesis [21–23]. In the lung, it seemed to be associated with obesity, intrinsic airway hyperresponsiveness, increased inflammatory and fibrotic gene expression [24]. SAA was recently proposed as a biomarker of IPF [25].

The aim of this study was to investigate inflammatory/fibrotic biomarkers, including biochemical analytes, acute phase proteins and KL-6 to determine whether single mediator or panels of mediators could be useful to stratify patients into three distinct domains: sarcoidosis, IPF and cHP.

## Materials and methods

### Study design and population

A total of 163 ILD patients (88 male (54%), 75 female (46%)) monitored at Siena Regional Centre for Sarcoidosis and other Interstitial Lung Diseases were enrolled in the study. Diagnosis of sarcoidosis, IPF and cHP was according to international criteria. The population included 97 patients with sarcoidosis; 24 of whom were selected for biomarker study. All patients underwent lung function tests, chest X-ray, high-resolution computed tomography of the chest (HRCT), immunological evaluation and blood sampling. Patients were not in therapy at the time of blood sampling. Serum samples were stored at − 80 °C until analysis. The sarcoidosis and cHP patients all had chronic persistent forms monitored at our referral centre; any patients with acute or subacute variants, including Lofgren syndrome and acute lymphocytic HP, were excluded. Biochemical data, including C reactive protein (CRP), red blood cell count (RBC), haemoglobin (Hb), white blood cell count (WBC), haematocrit (HCT), platelets (PLT), creatinine, urea, bilirubin, glutamic aspartate aminotransferase (AST), alanine aminotransferase (ALT), total cholesterol concentrations, high- and low-density lipoprotein (HDL, LDL) and triglycerides, were also recorded. The data was entered in a specific database. Patients were all Caucasians and gave their written informed consent to participation in the study, which was approved by our Local Ethics Committee (CEAVSE code number 180712).

### Lung function tests

The following lung function measurements were recorded according to ATS/ERS standard parameters using a Jaeger body plethysmograph with corrections for temperature and barometric pressure: forced expiratory volume in the first second (FEV1), forced vital capacity (FVC) and diffusing capacity of the lung for carbon monoxide (DL_CO_). All parameters were expressed as percentages of predicted values.

### Assay of serum SAA and KL-6 assay

Serum SAA was measured by enzyme linked-immuno-sorbent assay (ELISA kit, Invitrogen Corp.) following the manufacturer’s instructions. Concentrations were read at 450 nm with a Victor X4 fluorimeter (Perkin Elmer, Inc.). Concentrations of SAA were expressed in nanogram per millilitre.

Krebs von den Lungen-6 was measured in serum by KL-6 reagent assay (Fujirebio Europe, UK). The principle of the assay is agglutination of sialylated carbohydrate antigen in samples with KL-6 mAb by antigen–antibody reaction. The change in absorbance reflects KL-6 concentrations. KL-6 concentrations in samples were expressed in units per millilitre. The serum cut-off levels was 500 U/ml [9, 26, 27].

### Statistical analysis

The results were presented as means and standard deviations (SD) or as medians and inter quartiles (25th and 75th percentiles) for continuous variables. Data distributions were non-normal. The one-way ANOVA non parametric test (Kruskal-Wallis test) and Dunn test were performed for multiple comparisons. The chi-squared test was used for categorical variables, as appropriate. We also performed a logistic regression to compare parameters between with sarcoidosis and other ILD patients and between IPF and other ILD patients. We also performed a similar analysis with IPF as dependent variable against cHP. Associations are indicated as odds ratios (ORs) with 95% confidence intervals. We also assessed the validity of variables used to distinguish IPF and cHP by areas under curves (AUC) in the receiver operating characteristic (ROC). Sensitivity, specificity and positive and negative predictive values (PPV and NPV, respectively) were calculated for cut-offs of the different variables. The Youden index (*J* = max (sensitivity + specificity-1)) was used to establish the best cut-offs for diagnosis. A similar analysis was performed to test significant variables in the different diagnosis of sarcoidosis against the other ILDs and IPF against the other ILDs. A *p* < 0.05 was considered statistically significant. When performing several pairwise comparisons on the same variable, a Bonferroni correction for the *p* value was adopted. Spearman’s rank correlation was used to establish the relationship between analyte concentrations in each study group. Statistical analysis and graphic representation of the data was performed by GraphPad Prism 8.0 software.

## Results

### Study population

Demographic data (including sex, age and smoking habits) is reported in Table 1. As expected, patients with IPF and cHP were prevalently male ex-smokers, over 65 years of age, whereas sarcoidosis patients were prevalently young females who had never smoked.

**Table 1:** Demographic data and smoking habits of the population

	Sarcoidosis (*n* = 97)	IPF (*n* = 40)	cHP (*n* = 26)	*P* values
Gender (M/F)	39/58	33/7	16/10	0.002
Age (mean ± SD)	55 ± 10	69 ± 9	67 ± 9	0.001
Smoking habits (current/never/former)	10/75/12	0/35/5	0/14/12	0.003

### Comparative analysis of lung function tests

Lung function test parameters are reported in Table 2. Comparisons showed higher FVC (%), FEV1 (%) and DLCO (%)in sarcoidosis patients than in patients with IPF and cHP (*p* = 0.002; *p* < 0.001; *p* < 0.001, respectively).

**Table 2:** Pulmonary function tests and biochemical analytes. Data were expressed as median (interquatile range)

	Sarcoidosis (*n* = 24)	IPF (*n* = 40)	cHP (*n* = 26)	*P* values
PFTs:				
FEV1 (%)	97 (90–104)	73 (63–92)	78 (64–91)	< 0.0001
FVC (%)	106 (91–115)	70 (54–94)	74 (62–88)	0.002
DLCO (%)	76 (67–85)	44 (35–54)	58 (46–67)	< 0.0001
KL-6 (U/ml)	537 (300–1332)	2062 (1337–3864)	1146 (707–2431)	< 0.0001
SAA (ng/ml)	4370 (3612–6379)	7031 (5590–7454)	4022 (3469–4615)	< 0.0001
Biochemical data:				
CRP (mg/dl)	0.14 (0.10–0.24)	0.26 (0.17–0.62)	0.35 (0.15–0.91)	0.04
RBC(10^6^/mm^3^)	4.7 (4.4–4.9)	4.9 (4.5–5.1)	5 (4.4–4.9)	ns
Hb (g/dl)	13.9 (12.5–14.5)	14.4 (13.6–15.2)	14 (13.5–15.2)	ns
WBC(10^3^/mm^3^)	5.7 (5.3–7.3)	8 (6.8–9.3)	7 (6.2–9)	0.004
HCT (%)	42.9 (38.9–44)	65 (54.5–75.5)	43 (41.2–46-6)	ns
PLT (10^3^/mm^3^)	248 (215–303)	214 (182–254)	243 (199–366)	ns
Creatinine (mg/dl)	0.75 (0.7–0.86)	0.9 (0.7–1)	1 (0.78–0.94)	0.006
Urea (mmol/l)	3.5 (2.9–4.1)	3.3 (2.8–4)	3.8 (3.5–4.6)	ns
Bilirubin (mg/dl)	0.4 (0.4–0.7)	0.5 (0.4–0.6)	1 (0.4–0.6)	ns
AST (U/l)	19 (17–22)	19 (17–22)	18 (17–25.5)	ns
ALT (U/l)	18.5 (15.2–22)	15 (12.5–18.2)	17 (13–22.5)	ns
Cholesterol (mg/dl)	175.5 (165.5–200)	188.5 (168.5–203.5)	200 (178.5–223.7)	ns
HDL (mg/dl)	49 (45–53)	52.5 (45–66)	53 (48–60)	ns
LDL (mg/dl)	115 (95–140)	112.5 (90.7–150)	110 (94–126)	ns
Triglycerides (mg/dl)	100 (74–121.5)	113 (96.7–124.5)	105 (78–125)	ns

Direct comparisons between sarcoidosis and IPF and sarcoidosis and cHP parameters revealed differences in FEV1 (%), FVC (%) and DLCO (%) (*p* < 0.001), whereas direct comparison of IPF and cHP parameters only showed a significant difference for DLCO (%) (*p* = 0.03).

### Comparative analysis of laboratory variables

Clinical biochemistry parameters are reported in Table 2. Multiple comparison analysis showed significant differences in CRP (*p* = 0.04), WBC (*p* = 0.004) and creatinine concentrations (*p* = 0.04). In particular, CRP values resulted decrease in Sarcoidosis patients than HP (*p* = 0.005) and IPF (*p* = 0.00007). Also, WBC resulted statistical decrease in Sarcoidosis patients than HP (*p* = 0.003) and IPF (*p* = 0.0007). Creatinine concentrations (*p* = 0.01) and HDL cholesterol (*p* = 0.04) were decrease in sarcoidosis patients than cHP and IPF patients. IPF and sarcoidosis patients showed differences in CRP (*p* = 0.01), Hb (p = 0.01), HCT (*p* = 0.02), WBC (*p* < 0.001), PLT (*p* = 0.02), creatinine (*p* = 0.02) and GPT (*p* = 0.03) values. IPF and cHP patients showed differences in urea concentrations and RBC count (*p* < 0.05). No other significant differences were found.

### Comparative analysis of SAA and KL-6

Multiple comparison analysis showed differences in KL-6 and SAA concentrations (*p* < 0.0001). Interestingly, KL-6 concentrations were significantly different between sarcoidosis and IPF (*p* < 0.0001), sarcoidosis and cHP (*p* = 0.02) and IPF and cHP patients (*p* < 0.0001). SAA concentrations were significantly different in IPF and sarcoidosis (*p* < 0.0001) and in IPF and cHP patients (*p* < 0.0001).

### Sarcoidosis discriminant panel

**Fig. 1:**
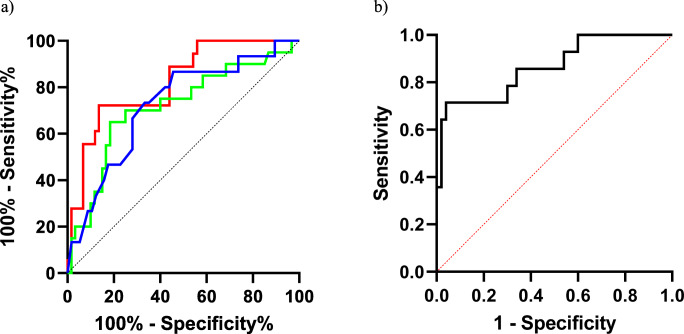
**a** ROC curve analysis of WBC (blue), KL-6 (red) ,and CRP (green) in classification of sarcoidosis diagnosis compared with other ILD. **b** Roc curve analysis of a panel of biomarkers

ROC curves were plotted to assess the diagnostic value of the parameters and to determine cut-off values with sufficient sensitivity and specificity in the ILD groups. Sarcoidosis patients showed the best areas under the curve (AUC) than patients with other ILDs for KL-6 (AUC = 0.82, 95%CI 0.71–0.92; *p* < 0.001), CRP (AUC = 0.72, 95%CI 0.57–0.86; *p* = 0.008) and WBC (AUC = 0.712, 95%CI 0.57–0.85 *p* = 0.0039) (Fig. 1a). In the logistic regression, sarcoidosis was tested as dependent variable, while KL-6, CRP and WBC were tested as independent variables. ROC curve analysis of model performance showed AUC 0.86 (95%CI 0.74–0.98; NPP (%): 89, PPP(%) 88.8; *p* < 0.0001) (Fig. 1b). KL-6 values below a cut-off of 803.5 IU/ml had 72.2% sensitivity and 86.4% specificity in discriminating sarcoidosis patients from other groups. CRP values below a cut-off of cut-off 0.15 mg/dl showed 73.3% sensitivity and 66.7% specificity in discriminating sarcoidosis patients from those of other groups. WBC counts below a cut-off of 616 (10^3^/mm^3^) showed 70% sensitivity and 75% specificity in discriminating sarcoidosis patients from patients of other groups.

### IPF discriminant panel

**Fig. 2:**
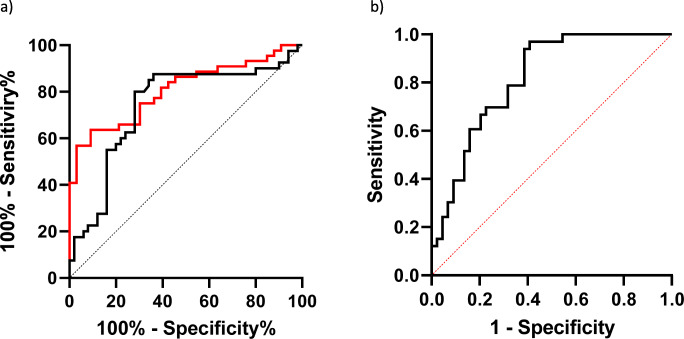
**a** ROC curve analysis of SAA (black) and KL-6 (red) in classification of IPF diagnosis compared with other ILD. **b** Roc curve analysis of a panel of biomarkers

In the IPF group, ROC curve analysis showed the best areas under the curve than for the other ILDs for KL-6 (AUC = 0.80, 95%CI 0.71–0.90; *p* < 0.0001) and SAA (AUC = 0.69, 95%CI 0.61–0.77, *p* < 0.0001) (Fig. 2a).

The logistic regression model with IPF as dependent variable and KL-6 and SAA as independent variables showed AUC 0.81 (95%CI 0.72–0.90; NPP (%):74, PPP (%) 74.1; *p* < 0.0001) (Fig. 2b). KL-6 values below a cut-off of 1678 IU/ml showed 75% sensitivity and 69.7% specificity in discriminating IPF patients from other groups. SAA values above a cut-off of 54.147 ng/ml showed 80% sensitivity and 72% specificity in discriminating IPF patients from those of other groups.

**Fig. 3:**
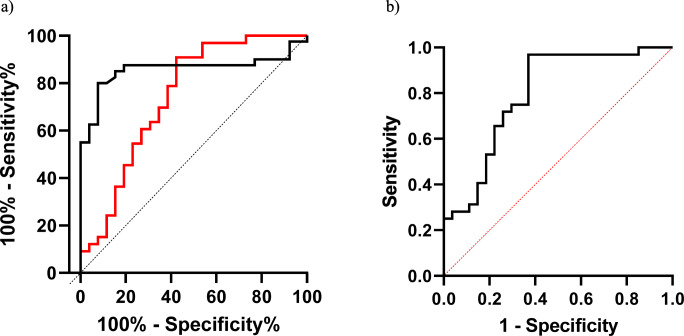
**a** ROC curve analysis of SAA (black) and KL-6 (red) in classification of IPF diagnosis compared with CHP. **b** Roc curve analysis of a panel of biomarkers

As IPF and cHP are respiratory disorders that frequently require differential diagnosis, we compared the parameters of the IPF and cHP cohorts. ROC curve analysis showed the best areas under the curve or KL-6 (AUC = 0.74, 95%CI 0.60–0.87; *p* < 0.0001) and SAA concentration (AUC = 0.85, 95%CI 0.76–0.95, *p* < 0.0001) (Fig. 3a). The logistic regression model with IPF as dependent variable and KL-6 and SAA as independent variables showed AUC 0.79 (95%CI 0.67–0.91; NPP (%)66.6, PPP (%) 75.9; *p* < 0.0001) (Fig. 3b). KL-6 values above a cut-off of 2206 IU/ml showed 60% sensitivity and 73% specificity in discriminating IPF patients. SAA values above a cut-off of 53,971 ng/ml showed 80% sensitivity and 92% specificity.

### Correlation analysis

Analysing each group separately, SAA and WBC were directly correlated (*r* = 0.62, *p* = 0.004) in the sarcoidosis group. In the IPF group, SAA was correlated with creatinine (*r* = −0.64, *p* < 0.0001) and CRP (*r* = 0.38; *p* = 0.02). In the cHP group, direct correlations were observed between KL-6 and HCT (*r* = 0.45, *p* = 0.03) and between KL-6 and RBC (*r* = 0.47, *p* = 0.025). CRP also showed a correlation with total cholesterol (*r* = −0.5, *p* = 0.03) and WBC count (*r* = 0.49, *p* = 0.01).

## Discussion

In this study, different combinations of biomarkers were analysed in an attempt to differentiate sarcoidosis, IPF and cHP patients. A diagnosis of sarcoidosis was associated with values of three different parameters: KL6, WBC and CRP, enabling this granulomatous inflammatory disorder to be distinguished from the fibrotic lung diseases IPF and cHP. The combination of these three biomarkers improved overall sensitivity and specificity with respect to the single parameters.

The role of these analytes in sarcoidosis patients has been widely investigated [25, 28, 29], although very few data were available concerning the comparison of sarcoidosis with other fibrotic lung diseases for a panel of biomarkers and based on the analysis of bronchoalveolar lavage fluid [30]. CRP is regarded as the major pro-inflammatory acute phase protein related to sarcoidosis [27]. In a very recent paper, Kobak et al. analysed lipid metabolism in sarcoidosis patients, reporting higher concentrations of CRP than in healthy controls [28]. Manuel Ramos-Casals et al. cited CRP among the most sensitive markers for confirming a diagnosis of sarcoidosis [26]. Our results, showing lower concentrations of CRP in sarcoidosis patients than in patients with IPF and CHP, may be useful to exclude other causes of ILD in the diagnostic workup of sarcoidosis.

Concerning KL-6, this protein has already been reported as a potential biomarker of fibrotic lung involvement in sarcoidosis and shows an inverse correlation with DLco [29, 31]. Since our sarcoidosis population included patients with mild lung involvement, lower KL-6 concentration than in IPF and cHP patients was expected.

In our population a reduction in WBC count was associated with sarcoidosis: lymphopenia is a common feature in sarcoidosis patients but not in other ILDs [32, 33]. It is due to recruitment of these immune cells in granulomas of the lungs or other affected organs [30, 34].

In our study, the combined use of SAA and KL-6 was significantly associated with a diagnosis of IPF. The role of biomarkers in the diagnosis of ILDs is generally controversial, as no indicator has proved to be sufficiently accurate [35]. In the literature, KL-6 is more associated with the evolution and prognosis of fibrotic lung disease than with diagnosis [21, 36]. The clinical, radiological and histopathological overlap of different ILDs makes it crucial to have reliable biomarkers for a personalized approach to these patients. Since single molecules have poor performance, combinations of biomarkers seemed worth investigating [37–39].

On the basis of our results, we propose a combination of biomarkers for discriminating IPFs from other ILDs, especially cHP. The panel showed good accuracy, specificity and sensitivity.

These results were quite unexpected: the role of SAA in fibrotic ILDs is largely unexplored as it has been considered a key molecule in granuloma formation and perpetuation [40–42]. However, in a previous study, we demonstrated higher serum concentrations of SAA in IPF patients than in healthy controls [41]. The diagnostic performance of SAA and KL-6 reported in our study is interesting and important, particularly the fact that when the cohort was restricted to IPF and cHP patients, the model maintained the same accuracy, with good specificity and sensitivity in discriminating these two diseases. IPF and cHP have hither to been indistinguishable and often misdiagnosed [36]. This non-invasive combination of biomarkers may further stimulate multidisciplinary discussion in the field of ILDs.

In conclusion, the combination of serum biomarkers proposed here offers insights into the pathobiology of ILDs and will improve diagnostic accuracy. Once validated in a larger cohort, the proposed panels of biomarkers will be useful in the clinical management of ILDs.

## Abbreviations

ILD: Interstitial lung diseases
IPF: Idiopathic pulmonary fibrosis
cHP: Cronic hypersensitivity pneumonitis
FVC: Forced vital capacity
FEV1: Forced expiratory volume in the first second
DL_CO_: Diffusing capacity of lung for carbon monoxide
UIP: Usual interstitial pneumonia
KL-6: Krebs von den Lungen 6
SAA: Serum amyloid A
CRP: C reactive protein
RBC: Red blood cell count
Hb: Haemoglobin
WBC: White blood cell count
HCT: Haematocrit
PLT: Platelets
AST: Aspartate aminotransferase
ALT: Alanine aminotransferase
HDL: High-density lipoprotein
LDL: Low-density lipoprotein
AUC: Areas under the curve
HRCT: High-resolution computed tomography
PFT: Pulmonary function test
